# Effect of Polymers and Permeation Enhancers in the Release of Quetiapine Fumarate Transdermal Patch through the Dialysis Membrane

**DOI:** 10.3390/polym14101984

**Published:** 2022-05-13

**Authors:** Mullaicharam Bhupathyraaj, K. Reeta Vijaya Rani, Sathvik B. Sridhar, Javed Shareef, Sabin Thomas, Nirmala Halligudi, Anbazhagan Sockalingam, Kiruba Mohandoss, Shyam Sundar

**Affiliations:** 1 College of Pharmacy, National University of Science and Technology, Muscat 130, Oman; nirmalahalligudi@nu.edu.om; 2 Surya School of Pharmacy, Vikravandi, Villupuram 605652, Tamilnadu, India; reeta_rani07@yahoo.co.in (K.R.V.R.); sanbubiochem@gmail.com (A.S.); 3 RAK College of Pharmaceutical Sciences, RAK Medical, and Health Sciences University, Ras Al Khaimah P.O. Box 11172, United Arab Emirates; sathvik@rakmhsu.ac.ae (S.B.S.); javedh@rakmhsu.ac.ae (J.S.); 4 School of Pharmacy, College of Pharmacy & Nursing, University of Nizwa, Nizwa 616, Oman; sabin@unizwa.edu.om (S.T.); asundar@unizwa.edu.om (S.S.); 5 Shri Sathya Sai Medical College and Research Institute, Thiruporur, Chengalpet 603108, Tamilnadu, India; mkirupa@gmail.com

**Keywords:** schizophrenia, quetiapine fumarate, percutaneous release, transdermal patch

## Abstract

Quetiapine Fumarate is potent, and the daily therapeutic dose can be delivered easily across the skin with the help of permeation enhancers. Quetiapine Fumarate-loaded transdermal patches were prepared by solvent evaporation technique. Various formulation parameters, excipients, and their combinations were optimized to get thin, translucent, smooth, stable, and high permeable character patches. A total number of 10 formulations were prepared. All formulations were subjected to various physicochemical evaluations. Three different formulations were prepared and F1, F2, and F3. Various physicochemical studies were carried out and found no significant difference between the three batches. The in vitro release study showed 74.29%, 82.73%, and 77.27%, respectively, up to 24 h. From the results, F2 has been selected as an optimized formulation and evaluated for skin irritation test. The results revealed that there is no irritation produced. The stability study results showed that there is no significant change from its initial nature till the period of three months in both temperatures. Quetiapine Fumarate Transdermal Patch F2 has achieved the goal of extended-release, cost-effectiveness, lowering the dose and frequency of drug administration, and thus may improve patient compliance.

## 1. Introduction

According to WHO, schizophrenia affects about 24 million people worldwide. It is a treatable disorder, with the treatment being more effective in its initial stages. More than 50% of people with schizophrenia are not receiving appropriate care. A total of 90% of people with untreated schizophrenia are in developing countries [1,2,3]. Available oral dosage form has severe hepatic first-pass metabolism, resulting in much less oral bioavailability [4]. The major metabolic pathways are sulfoxidation to the sulfoxide metabolite and oxidation to the parent acid metabolite; both metabolites are pharmacologically inactive [5]. Quetiapine is an atypical antipsychotic drug used for the treatment of schizophrenia and bipolar disorder. Its bioavailability is only 9% and it has a short half-life (6 h) due to inherent hepatic metabolism. To address these issues, a matrix-type transdermal patch of Quetiapine Fumarate was developed by using the polymers Poly Vinyl Pyrollidone K30 and Hydroxy Methyl Cellulose K 100. Quetiapine is categorized as a serotonin antagonist, which binds to 5-HT 2A/2C receptors. It has a strong affinity to several dopaminergic receptors but exerts weak antagonism for the D2 receptor, which is responsible for modulating the neuroleptic activity [3]. Quetiapine Fumarate formulations produce dose-dependent side effects like extrapyramidal effect akinesia (inability to initiate movement), akathisia (inability to remain motionless), dyskinesia (disorder resulting in involuntary, repetitive body movements), diatonic symptoms (sustained muscle contractions), and neuroleptic malignant syndrome (muscle rigidity, autonomic instability). The therapeutic equivalent dosage for the transdermal delivery of certain compounds can be significantly less than the corresponding oral dosage, thereby potently reducing the dose-related side effects. Further, a gradual reduction in the dose of the drug could be achieved by simple replacement of the patches containing various doses of the drug [6]. This dosage adjustment may alleviate the side effects that occur after the immediate termination of antipsychotics. The possible overdoses that often occur in the oral route can also be avoided by the transdermal administration of antipsychotics. The patient could be maintained in a well-controlled condition (by maintaining the plasma drug concentration). The transdermal patch can be applied to uncontrolled or calm patients. Quetiapine Fumarate is potent, and the daily therapeutic dose can be delivered easily across the skin with the help of penetration enhancers [7,8,9]. Concerning the commercial view, the market for transdermal products is ever-increasing (USD 31.5 billion in the year 2015) and approximately 13 active ingredients are approved for use globally [10]. This scenario of the transdermal market shows the commercialization potential of the developed systems in the future.

## 2. Materials and Methods

### 2.1. Materials

Quetiapine Fumarate was obtained as a gift sample from Octopus Pharmaceuticals, Chennai, India. HPMC K 100 was obtained from Hi-media laboratories, Mumbai, India. Poly Vinyl Pyrrolidone K 30 was procured from BASF limited, Rosenberg, Germany. Linalool and 1,8-Cineole were procured from Alfa Aesar, Lancaster, UK. DMSO and Glycerin have been purchased from Merck Limited, Vikroli East, Maharashtra, Mumbai. All other chemicals were of analytical grade.

### 2.2. Methods

#### 2.2.1. Compatibility by FTIR Spectroscopy

Drug-Excipient compatibility was carried out by FT-IR analysis [11]. Initially, the IR spectrum of pure drug, Quetiapine Fumarate, and individual spectrum of PVP, HPMC, glycerin, cineole, linalool, and DMSO were obtained. After that, various admixtures of the drug with other excipients were prepared and the IR Spectra were obtained in KBr pellets using a Perkin Elmer model spectrum (Thane, Maharashtra) in the range 4000–400 cm^−1^.

#### 2.2.2. Partition coefficient

The partition coefficient was done using n-octanol as the oil phase and phosphate buffer pH 7.4 as the aqueous phase. The two phases were mixed in equal quantity and 10 mg of the weighed amount of drug was added. Then, these were flooded on a mechanical shaker for 2 h. The saturated phases were separated by a separating funnel and an equal volume of both phases n-octanol and phosphate buffer were taken in a conical flask and then analyzed for respective drug controls. The partition coefficient of drug Po/w was calculated by the following formula [12].

Partition coefficient [Po/w] = Coil/Cwater (at equilibrium)

Preparation of transdermal patch of Quetiapine Fumarate

Preliminary trial batches (T1–T10) of Quetiapine Fumarate matrix type transdermal patches were prepared by solvent evaporation method [13] using a Petri plate. The trial batches were composed of PVP K 30 and HPMC K 100 in 1:1, 1:2, 1:3, 1:4, 1:5, 1:6, 1:7, 1:8, 1:9, and 1:10 ratios, respectively (in all formulations the PVP K 30 quantity was kept constant). The polymer solution was prepared using distilled water and was stirred well to get a homogenous mixture using a magnetic stirrer. A specified percentage of glycerin was added as a plasticizer. A weighed quantity of the drug was dissolved in the polymeric solution and finally made up to the required volume using water and kept aside a few hours to remove the air bubbles. Then, the solution was poured into the Petri dish coated with aluminum foil and dried at room temperature for 24 h for solvent evaporation and an inverted funnel was kept over the Petri dish to control the evaporation of the solvent. The patches were removed by peeling and were cut into squares carrying 20 mg of Quetiapine Fumarate/patch area, which were kept in a desiccator for physicochemical evaluations and percentage drug content. Finally, the prepared patches were visually compared with the commercially available transdermal patch of matrix diffusion type (Nu Patch—Zydus Cadila, Ahmedabad, India).

### 2.3. Evaluation of Preliminary Trial Batches

#### 2.3.1. Water Vapor Transmission (WVT) Rate

A glass vial is used as a transmission cell, in which calcium chloride was placed, acting as a desiccant. A patch that is to be evaluated was placed over the cell. This cell was weighed and placed in a desiccator, which is filled with Potassium Chloride solution (saturated solution) to maintain the 84% RH. Glass vial was removed from the desiccator and reweighed after 24 h for a period of 72 h. The WVT rate was determined using the following formula [14].

$$\mathrm{WVT}=\frac{\mathrm{Final}\;\mathrm{Weight}-\mathrm{Initial}\;\mathrm{Weight}\;\times 100}{\mathrm{Exposure}\;\mathrm{time}\;\times \;\mathrm{Area}\;\mathrm{of}\;\mathrm{patch}},$$

#### 2.3.2. Percentage Moisture Content

The prepared patches were weighed individually and kept in a desiccator containing fused calcium chloride at room temperature for 24 h. After 24 h, the patches were reweighed, and the percentage moisture content was determined using the following formula [15].

$$\mathrm{Percentage}\;\mathrm{moisture}\;\mathrm{content}\;=\frac{\mathrm{Initial}\;\mathrm{weight}-\;\mathrm{Final}\;\mathrm{weight}\;\times 100}{\mathrm{Final}\;\mathrm{weight}}.$$

#### 2.3.3. Percentage Moisture Uptake

The weighed patches were kept in a desiccator at room temperature for 24 h containing a saturated solution of potassium chloride to maintain 84% RH. After 24 h, the patches were reweighed and the percentage moisture uptake was determined using the following formula [16].

$$\mathrm{Percentage}\;\mathrm{moisture}\;\mathrm{content}\;=\frac{\mathrm{Final}\;\mathrm{weight}-\;\mathrm{Initial}\;\mathrm{weight}\times 100}{\mathrm{Initial}\;\mathrm{weight}}$$

#### 2.3.4. Thickness

The thickness of the patch was measured at three different points using a digital Vernier caliper and the average thickness was calculated [17].

#### 2.3.5. Weight Uniformity

For each formulation, three randomly selected patches were used. For the weight variation test, three patches from each batch were weighed individually and the average weight was calculated [18].

#### 2.3.6. Folding Endurance

The folding endurance of the patch was determined by repeatedly folding a small strip of the patch (2 cm × 2 cm) at the same place until it broke. The number of times the patch could be folded at the same place without breaking gave the value of folding endurance [19].

#### 2.3.7. Drug Content

The uniformity of drug content of the transdermal patch was determined, based on the dry weight of the drug and polymer used, using a UV/VIS spectrophotometer method [20]. A specified area (2.5 cm^2^) of the patch was cut and dissolved in 5 mL of phosphate buffer pH 7.4. Then the solution was transferred to a volumetric flask and the volume was made up to 10 mL. Appropriate dilutions were made using phosphate buffer (pH 7.4), filtered, and analyzed for drug content at 271 nm by using a UV spectrophotometer (Shimadzu-1700, San Francisco, CA, USA).

#### 2.3.8. Incorporation of Permeation Enhancers and Evaluation of Permeation Enhancers Incorporated Trial Batches

After the selection of the best formulation based on the physical properties and drug content evaluations, the selected formulation was again made into three different batches (F1, F2, and F3) with the incorporation of three permeation enhancers (Linalool, 1,8-cineole, and DMSO) in suitable proportions and evaluated for physical parameters such as Weight variation, Percentage Moisture uptake, Percentage moisture content, Thickness, and Percentage drug content.

#### 2.3.9. In Vitro Permeation Study Using Dialysis Membrane

In vitro permeation studies were carried out using Keshary–Chien diffusion cells. The dialysis sac was previously soaked for 24 h in phosphate buffer 7.4. The patches have adhered to the barrier membrane (dialysis membrane) and the sac is tied firmly to the donor compartment of the Keshary–Chien diffusion cell, the receptor compartment of which is filled with 100 mL phosphate buffer 7.4. The total setup was placed on a thermostatically controlled magnetic stirrer set at 37 ± 2 °C. The content of the diffusion cell was stirred at a constant speed (100 rpm). Samples were withdrawn (1 mL) at predetermined time intervals and replaced with the same amount of distilled water to maintain the sink condition. The samples were analyzed for drug content using a UV spectrophotometer at 271 nm. The permeation study was carried out for 24 h [21].

#### 2.3.10. Skin Irritation Study of Quetiapine Fumarate Transdermal Patch

The irritation study was performed on three male healthy rabbits (average weight: 1.43 kg) according to the Draize modified scoring technique [22]. The dorsal surface (50 cm^2^) of the rabbit was cleaned and the hair was removed by shaving. The skin was cleaned with rectified spirit. Representative patches were placed over the skin with the use of adhesive tape and were removed after a period of 1 h, 24 h, 48 h, and 72 h and observed for any skin reactions of erythema and edema. The average of 1–72 h readings represents the final score. Ethical clearance was obtained from the institutional animal ethical committee formed for this purpose (IAEC number in Skin irritation study method PCP/IAEC/001/2018).

The Primary Dermal Irritation index (PDI) was calculated from the average scores of the skin reactions. The skin reactions were scored as follows 0—no erythema and edema, 1—very slight erythema and very slight edema, 2—well-defined erythema and slight edema, 3—moderate to severe erythema and moderate edema, 4—severe erythema and severe edema [23,24,25,26].

#### 2.3.11. Stability Study

The patch was packed in aluminum foil and tested at accelerated conditions at temperatures of 40 ± 2 °C and 75 ± 5% RH for 3 months [27]. Patches were assessed for visual appearance, color, texture, drug content, and release profile [28].

## 3. Results

FT-IR spectral analysis of Quetiapine Fumarate and formulations F2 were obtained. The observations concluded that there is no interaction between the drug and other excipients and the results are given below.

The FTIR spectral analysis of Quetiapine Fumarate alone showed the principal peaks at wave numbers of different Functional groups.

Wave Number in cm  Functional groups

3784.09  O–H Stretching

3064.45  C–H Stretching

2086.41  C=C Stretching

1417.06  C–H Deformation

1246.63  C–O Stretching

987.89, 779.05 C–H out of plane bending

The FTIR spectral analysis of formulation F2 alone showed the principal peaks at wave numbers of different Functional groups.

Wave Number in cm^−1^   Functional groups

3785.37   O–H Stretching

3310.74   =C=H Stretching

2934.92   C–H Stretching

1602.87   C=O Stretching

1416.87   O–H Deformation

The preliminary trial batches, composed of drug Quetiapine Fumarate, polymers PVP K 30, HPMC K 100, plasticizer glycerin, and solvent distilled water, are given in Table 1.

Various physicochemical properties of Preliminary trial formulations such as WVTR, % Moisture uptake, % moisture content, thickness, folding endurance, and % drug content were evaluated for selection of the best formulation and the results are represented in Table 2.

Based on the physicochemical properties of the preliminary trial formulations, T5 has been selected as an optimized formulation and selected for incorporation of permeation enhancers. The composition of permeation enhancers incorporated T5 formulation and was divided into three batches, namely F1, F2, and F3. The composition is given in Table 3.

The physical properties such as weight, % moisture uptake, % moisture content, thickness, and % drug content were evaluated and the results are represented in Table 4.

**Figure 1:**
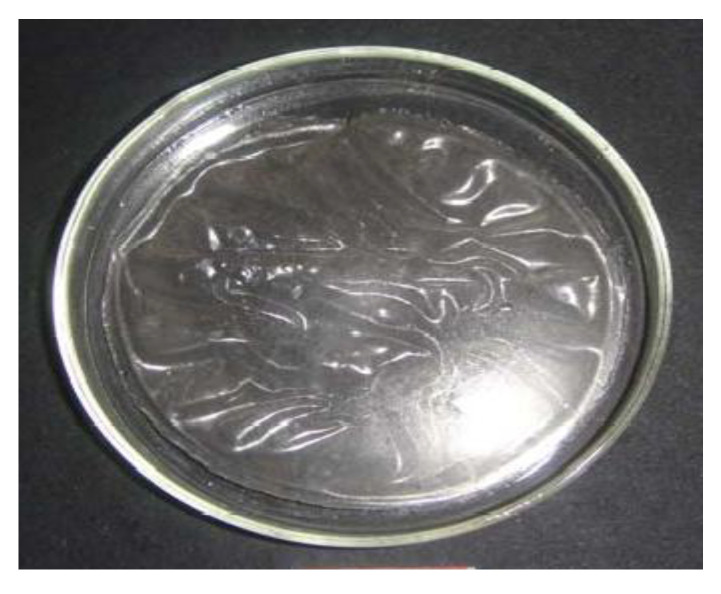
Transdermal patch of F2 formulation.

Hence, F2 showed better physical and in vitro permeation results, and was selected as the best formulation. The photo of formulation F2 is represented in Figure 1.

**Figure 2:**
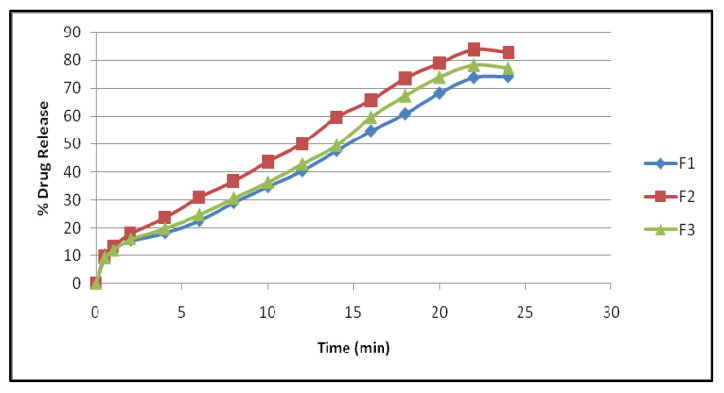
Comparative in vitro permeation profile of F1, F2, and F3.

The comparative in vitro permeation study was carried out for F1, F2, and F3. The formulation F2 shows better release compared to F1 and F2 and the results are explored in Table 5 and Figure 2.

The optimized formulation F2 has been subjected to a skin irritation study and the skin reactions were scored. The average score and PDI calculated for the patches are given in Table 6 and Table 7.

Total: 0/0 = 0

Mean: 0/4 = 0

PDI for 1, 24, 48 and 72 h = 0/4 = 0

Total: 1/0 = 0

Mean: 1/4 = 0.25

PDI for 1, 24, 48 and 72 h = 0.25/4 = 0.0625

**Figure 3:**
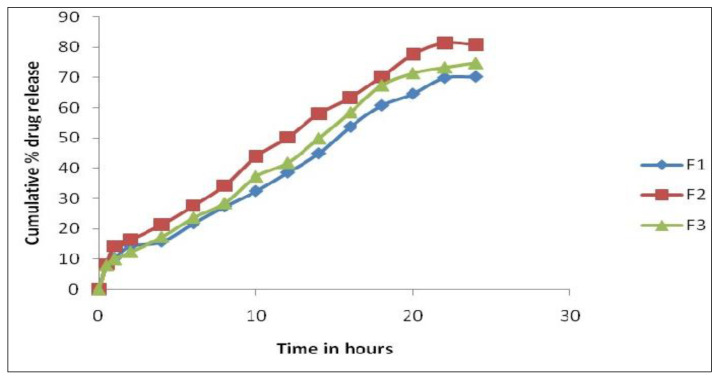
Cumulative percentage of in vitro permeation study of F2 formulation at 40 ± 2 °C and 75 ± 5% RH.

Further, formulation F2 was subjected to accelerated stability studies. The physicochemical parameters and cumulative in vitro permeability results at the end of the first, second, and third months are interpreted in Table 8 and Table 9 and Figure 3.

## 4. Discussion

Solvent evaporation was used to develop a transdermal patch containing quetiapine fumarate. These thin, translucent, smooth, stable, and very permeable patches were created by combining many formulation parameters. Each batch appears to be identical and contains no apparent fractures.

It was evident from the FTIR graphs that the major functional groups of pure Quetiapine Fumarate were not affected by polymer interaction or preparation methods. The drug’s partition coefficient was 2.6 in n-octane/phosphate buffer pH 7.4, indicating lipophilicity. Higher valued compounds are extremely lipophilic and stay dissolved in the stratum corneum.

A mixture of PVP K 30 and HPMC K 100 was shown to be the most effective patch-producing polymer. The overall polymer concentration was kept between 0.2 and 0.4 g per patch to provide folding durability, slimness, and an appropriate thickness.

The critical analysis of the plasticizer includes the plasticizer glycerol, which was applied at a concentration of 30% *w*/*w* of the total polymer weight, and was able to generate a flexible patch without having a significant impact on their releasing property.

The patch loses its flexibility and becomes stiff if the amount of plasticizer, glycerol, is larger than 0.1 mL (i.e., 30% *w*/*w* of polymer), therefore the above two factors were adjusted to make a proper patch. This plasticizer penetrates the polymer particles and softens them. Latex coalescence and patch formation are aided by this softening.

It becomes thick and breaks when the polymer concentration surpasses 0.2–0.4 g/mL. At 50 mg PVP, the patch becomes too thin and no longer fulfills the physicochemical requirements.

The slope of the Korsmeyer–Peppas equation indicates that ‘n’ is smaller than 0.5. This indicates that the transdermal patch released the medicine via a fickian (diffusion-controlled) transport mechanism, presumably as a result of a polymer that regulates drug diffusion across the polymeric matrix.

The patch’s medication release followed the zero-order equation. The correlation between the Hixson–Crowell and motored cube root equations is poor.

The improved formulation’s skin irritation was also tested. The mice in the F2 skin irritation investigation showed no erythema or edema after 1 h, 24 h, 48 h, and 72 h. So, it is safe to use.

The formulation T5 had the greatest WVTR, % moisture absorption, % moisture content, thickness, folding endurance, and % drug content of the 10 testing formulations. This may be a result of both polymers’ hydrophilicity.

There was no discernible change in the drug content between the patches of formulation T1 to T8. The results suggested that the approach utilized to manufacture a patch with a homogeneous medication content and a small number of patch variables was effective. This refers to the uniform distribution of the medicine throughout the patch production process.

The chosen formulation was compared to a commercial transdermal matrix diffusion patch. Three-month stability tests were conducted. Visual appearance, color, texture, drug content, and drug release were investigated. The results demonstrated no significant change in visual appearance, color, or texture during the period of three months. Accelerated heating lowered the drug content to 95.34%. The accelerated temperatures have no effect on the release kinetics.

Data analysis of the work includes the following:

A natural and synthetic permeation enhancer, linalool 1,8-cineole, was added to the T5 formulation to test its capacity to penetrate the dialysis membrane (three formulations were developed: namely, F1, F2, and F3). Physical factors such as weight fluctuation, thickness, moisture content, moisture absorption, folding endurance, and medication content are also evaluated.

This F1, F2, and F3 weighed 345, 380, and 350 mg. The three formulations were 0.48 mm, 0.53 mm, and 0.47 mm thick. Like the commercial version, this F2 formulation was optimally thick.

F2 had the highest % moisture content and absorption, resulting in a more flexible, easy-to-handle, and smooth patch.

F1–F3 showed a drug content of 98.86%, 99.48%, and 99.24%. There was no change. The 24 h in vitro release profile of F1–F3 exhibited a maximum release of 74.29%, 82.73%, and 77.27%, correspondingly.

The 90% release data were fitted to multiple kinetic models [29,30,31,32,33] to determine the mechanism and rate of drug release. There was an initial burst of the drug followed by a longer sustained release in the F2 formulation. After 24 h, F1 and F3 released little, however, F4 released in an erratic pattern.

This study helped to determine the polymer’s efficacy in controlled release testing.

We determined the efficacy of the dialysis membrane permeation enhancers to investigate them further in ex vivo and in vivo in an animal model investigation.

Future studies on the pharmacokinetic characteristics of the novel topical formulation of Quetiapine Fumarate can be done in conjunction with this investigation.

## 5. Conclusions

Based on the explanation above, it was ascertained that the F2 formulation of Quetiapine Fumarate Transdermal Patch, prepared using a 50:250 proportion of polymers PVP K30 and HPMC K 100, along with the natural permeation enhancer 1,8 cineol, was even more effective in terms of permeation and sustainability. A guideline for developing a non-invasive and sustained release drug delivery system for Quetiapine Fumarate for the successful treatment of schizophrenia is provided in this study endeavor. It is stable under storage settings for three months, with no obvious change in appearance, physical qualities, drug content, or percentage of drug release. The formulation is stable for three months. This formulation may be an alternative to the twice-daily oral conventional pills now available on the market. It has the potential to increase patient compliance, particularly in the case of recalcitrant patients.

## Figures and Tables

**Table 1:** Preliminary trial formulations.

S. No.	Quetiapine Fumarate (mg)	PVP K 30 (mg)	HPMC K 100 (mg)	Glycerin (mL)	Water (mL)
T1.	100	50	50	0.4	5
T2.	100	50	100	0.4	5
T3.	100	50	150	0.4	5
T4.	100	50	200	0.4	5
T5.	100	50	250	0.4	5
T6.	100	50	300	0.4	5
T7.	100	50	350	0.4	5
T8.	100	50	400	0.4	5
T9.	100	50	450	0.4	5
T10.	100	50	500	0.4	5

**Table 2:** Physicochemical properties of preliminary trial formulations.

Batch Code	WVTR 2 (g/c)	% MU	% MC	Thickness (mm)	Folding Endurance (n’s)	% Drug Content
T1	0.0182 ± 0.43	12.932 ± 0.94	2.813 ± 0.88	0.38 ± 0.52	192 ± 0.73	98 ± 0.11
T2	0.0291 ± 0.73	6.961 ± 0.77	3.749 ± 0.62	0.42 ± 0.76	145 ± 0.69	97 ± 0.23
T3	0.0149 ± 0.51	10.941 ± 0.82	1.423 ± 0.49	0.29 ± 0.82	162 ± 0.48	98 ± 0.53
T4	0.0281 ± 0.33	9.337 ± 0.61	2.938 ± 0.61	0.31 ± 0.69	202 ± 0.85	98 ± 0.71
T5	0.0441 ± 0.86	14.113 ± 0.06	6.231 ± 0.26	0.51 ± 0.57	214 ± 0.79	99 ± 0.22
T6	0.0301 ± 0.59	11.859 ± 0.27	3.882 ± 0.51	0.21 ± 0.94	154 ± 0.11	98 ± 0.84
T7	0.0221 ± 0.66	10.330 ± 0.84	1.391 ± 0.63	0.38 ± 0.48	198 ± 0.77	98 ± 0.68
T8	0.0182 ± 0.17	9.867 ± 0.49	3.830 ± 0.89	0.34 ± 0.22	172 ± 0.49	98± 0.53
T9	0.0159 ± 0.91	11.769 ± 0.63	2.670 ± 0.44	0.18 ± 0.61	182 ± 0.62	97 ± 0.71
T10	0.0281 ± 0.08	12.891 ± 0.38	1.462 ± 0.74	0.16 ± 0.38	196 ± 0.82	97 ± 0.44

WVTR—Water vapor transmission rate, MU—moisture uptake, MC—moisture content.

**Table 3:** Trial batches of optimized formulation with various permeation enhancers.

S. No.	Quetiapine Fumarate (mg)	PVP K 30 (mg)	HPMC K 100 (mg)	Glycerol (mL)	Water (mL)	Cineole (mL)	DMSO (mL)	Linalool (mL)
F1	10	50	250	0.4	5	-	-	0.3
F2	10	50	250	0.4	5	0.3	-	-
F3	10	50	250	0.4	5	-	0.3	-

**Table 4:** Physicochemical properties of optimized formulation with various permeation enhancers.

Code	Weight	% MU	% MC	Thickness (mm)	% Drug Content
F1	345 ± 3.47	13.543 ± 0.27	6.302 ± 0.15	0.48 ± 0.02	98.86 ± 1.03
F2	380 ± 4.09	14.134 ± 0.31	6.854 ± 0.18	0.53 ± 0.06	99.40 ± 1.76
F3	350 ± 3.12	13.321 ± 0.29	6.451 ± 0.16	0.47 ± 0.04	99.24 ± 1.34

**Table 5:** Comparative in vitro permeation study results of F1, F2, and F3.

Time (h)	% Drug Release ± S.D. *		
	F1	F2	F3
0	0	0	0
0.5	9.98 ±0.07	9.47 ± 0.02	9.12 ± 0.13
1	12.37 ± 0.04	13.37 ± 0.01	11.84 ± 0.11
2	15.37 ± 0.09	17.98 ± 0.07	15.95 ± 0.13
4	18.26 ± 0.05	23.68 ± 1.21	19.81 ± 0.16
6	22.65 ± 0.12	30.87 ± 0.09	24.71 ± 0.62
8	29.08 ± 0.09	36.62 ± 0.02	30.63 ± 0.17
10	34.74 ± 1.02	43.64 ± 0.08	36.29 ± 0.09
12	40.41 ± 1.05	50.19 ± 0.01	42.78 ± 0.15
14	47.63 ± 1.09	59.59 ± 0.05	49.46 ± 0.16
16	54.83 ± 1.03	65.67 ± 1.01	59.65 ± 0.98
18	60.98 ± 1.20	73.48 ± 0.08	67.35 ± 0.42
20	68.37 ± 1.09	78.93 ± 0.04	73.97 ± 0.50
22	73.92 ± 1.10	83.72 ± 1.21	78.29 ± 0.02
24	74.29 ± 1.12	82.73 ± 1.01	77.27 ± 1.01

* S.D. = standard deviation *n* = 3.

**Table 6:** Erythema and edema of the transdermal placebo patch.

Animal No.	Scores after Treatment with Placebo Patch without Drug after			
	1 h	24 h	48 h	72 h
**1**	0/0	0/0	0/0	0/0
**2**	0/0	0/0	0/0	0/0
**3**	0/0	0/0	0/0	0/0

**Table 7:** Erythema and edema of the selected (F2) formulation.

Animal No.	Scores after Treatment with a Drug-Loaded Patch after			
	1 h	24 h	48 h	72 h
**1**	1/0	0/0	0/0	0/0
**2**	0/0	0/0	0/0	0/0
**3**	0/0	0/0	0/0	0/0

**Table 8:** Physicochemical parameters of F2 formulation at 40 ± 2 °C and 75 ± 5% RH at the end of the first, second, and third months.

S. No.	Parameters	Room Temperature	40 ± 2 °C and 75 ± 5% RH
1.	Visual Appearance	Translucent	Translucent
	Initial	No change	No change
	1st month	No change	No change
	2nd month	No change	No change
	3rd month	No change	No change
2	Colour	Dull white	Dull white
	Initial	No change	No change
	1st month	No change	No change
	2nd month	No change	No change
	3rd month	No change	No change
3	Texture	Smooth	Smooth
	Initial	No change	No change
	1st month	No change	No change
	2nd month	No change	No change
	3rd month	No change	No change
4	Drug Content	No change	No change
	Initial	99.48	99.48
	1st month	99.09	98.92
	2nd month	98.48	96.65
	3rd month	97.97	95.34

**Table 9:** Cumulative percentage of the in vitro permeation study of F2 formulation at 40 ± 2 °C and 75 ± 5% RH.

Time (h)	Percentage of Drugs Released after Storage at the End of		
	1st Month	2nd Month	3rd Month
0	0	0	0
0.5	8.01	8.23	7.89
1	10.3	14.34	10
2	14.4	16.49	12.4
4	15.8	21.43	17.3
6	21.8	27.55	23.7
8	27.3	34.29	28.5
10	32.4	43.87	37.3
12	38.6	50.12	41.8
14	44.9	57.99	49.8
16	53.7	63.27	58.5
18	60.8	70.19	67.4
20	64.6	7.39	71.4
22	69.9	81.32	73.3
24	70.2	80.87	74.7

## Data Availability

Not applicable.
